# Assessment of meibomian gland morphology by noncontact infrared meibography in Shih Tzu dogs with or without keratoconjunctivitis sicca

**DOI:** 10.1111/vop.12645

**Published:** 2019-02-04

**Authors:** Yasunari Kitamura, Seiya Maehara, Tetsuya Nakade, Yukihiro Miwa, Reiko Arita, Hiroko Iwashita, Akihiko Saito

**Affiliations:** ^1^ Yakumo Animal Hospital Yakumo‐cho Japan; ^2^ Department of Small Animal Clinical Sciences, School of Veterinary Medicine Rakuno Gakuen University Ebetsu Japan; ^3^ Department of Ophthalmology Keio University School of Medicine Tokyo Japan; ^4^ Itoh Clinic Saitama Japan; ^5^ Triangle Animal Eye Clinic Tokyo Japan

**Keywords:** dog, keratoconjunctivitis sicca, meibomian gland, morphology, noncontact infrared meibography, Shih Tzu

## Abstract

**Objective:**

To investigate meibomian gland (MG) morphology by noncontact infrared meibography in Shih Tzu dogs with or without keratoconjunctivitis sicca (KCS).

**Procedures:**

Fourteen eyes of 12 Shih Tzu dogs (mean age of 10.7 years, range of 7‐13 years) presented to Yakumo Animal Hospital or Triangle Animal Eye Clinic from 2011 to 2017 with clinical signs and a Schirmer tear test (STT) result consistent with KCS (<10 mm/min) were examined. Twenty‐eight eyes of 16 Shih Tzu dogs (mean age of 12.4 years, range of 8 to 15 years) with a STT > 15 mm/min served as healthy controls. Both groups of dogs underwent routine slitlamp biomicroscopy followed by noncontact infrared meibography of the upper eyelid with both desktop‐type and mobile‐type systems.

**Results**

Meibography revealed morphological abnormalities of MGs in 13 eyes of 11 dogs with KCS. The abnormalities included gland shortening in 64% and gland dropout in 64% of the 14 eyes in the KCS group. Morphological changes were also observed in MGs of 16 eyes of 10 dogs in the control group. These changes included shortening in 46% and dropout in 17.8% of the 28 eyes in the control group. Dropout was significantly more common in eyes with KCS than in control eyes (*P < *0.01).

**Conclusions**

The frequency of MG abnormalities is increased in Shih Tzus with KCS compared with control animals. A reduced quality of the tear film associated with increased evaporation and reduced retention of tear fluid likely exacerbates the effects of a reduced tear volume in animals with aqueous deficiency.

## 1 INTRODUCTION

The tear film is composed of lipid layer and aqueous layer with soluble mucin, with the former being the most superficial and formed by lipids secreted by meibomian glands (MGs). The lipid layer thus decreases the evaporation of lacrimal fluid, prevents the washing away of tear fluid from the borders of the eyes, and facilitates smooth blinking. If the conjunctiva is not pigmented, the morphology of MGs can be assessed by observation of the gland orifices with a slitlamp; however, we have limited information by slitlamp examination. In the clinical setting, the development of noncontact infrared meibography that allows the noninvasive observation of MGs has greatly facilitated the evaluation of gland morphology in humans.^1^ The observation of MGs by noncontact infrared meibography is based on illumination of the tarsal plate with infrared light. The main abnormalities of MG morphology revealed by infrared meibography we considered include cystic dilatation, ductal openings occlusion or retroplacement, gland shortening (partial loss), and dropout (complete loss). Both desktop and portable noncontact infrared meibography systems are now available and are applied clinically to patients of all ages.^1^, ^2^ In addition to their clinical use, these meibography systems have been applied to studies of age‐related changes in MG morphology^1^ and of the relation between various eye diseases and MG abnormalities.^3^, ^4^, ^5^, ^6^, ^7^

In the field of veterinary ophthalmology, we have previously examined morphological changes in canine MGs using noncontact infrared meibography as well as performed histological analysis for areas in which the morphological changes were observed.^8^ We found that the prevalence of gland shortening and dropout identified by meibography tended to increase with age. Furthermore, the histopathologic findings for the sites of MG abnormalities included ductal dilatation and gland tissue breakdown. The morphological changes of MGs detected by meibography thus appeared to be related to histological findings. The relation between ocular surface disease and meibographic findings in dogs has remained unclear, however. We have therefore now examined quantitative changes in lacrimal fluid and morphological changes of MGs in dogs with keratoconjunctivitis sicca (KCS).

## 2 MATERIALS AND METHODS

The study sample included 14 eyes of 12 Shih Tzu dogs aged 7‐13 years (mean of 10.7 years) that presented at Yakumo Animal Hospital or Triangle Animal Eye Clinic between November 2011 and January 2017. The age, sex, and findings at initial examination for each animal are presented in Table 1. The 14 eyes were diagnosed with KCS on the basis of clinical symptoms including purulent mucinous discharge, conjunctival hyperemia, and keratitis as well as a Schirmer tear test (STT) (Schering‐Plough Animal Health) value of ≤10 mm/min. In addition, 28 eyes of 16 Shih Tzu dogs (aged 8‐15 years, with a mean of 12.4 years) without marked symptoms of the cornea and conjunctiva and with a STT > 15 mm/min were included as a control group. Ophthalmologic tests included an eye examination with a slitlamp, ocular tonometry, and fluorescein staining of the cornea. The morphology of MGs was observed from the conjunctival side, with the upper eyelid rotated outward, with the use of noncontact infrared meibography systems (Topcon BG‐4M and Japan Focus Meibopen). The lower eyelids were not examined because the strong pressure required to turn them outward could have caused pain to the animals. Shortening and dropout were scored using the following grades for upper eyelid: 0 (no loss or shortening of meibomian glands), 1(area loss or shortening was less than one third of the total meibomian glands), 2 (area loss or shortening was between one third and two thirds), 3 (area loss was more than two thirds). Signs of ocular inflammation (corneal vascularization, pigmentation) recorded if present. Corneal vascularization was classified in 4 grades as follows: 0(no vessel), 1(mild superficial vascularization visible with slitlamp), 2 (moderate superficial vascularization visible with naked eyes), 3 (severe vascularization of entire cornea). Corneal pigmentation was graded as follows 0 (no pigment), 1 (slight: partial pigmentation), 2 (marked: extensive pigmentation).

**Table 1:** The different breeds, gender, age, and eye condition of each dog investigated

	Age (years)	Gender	Eye	Schirmer (mm/min)	Condition	Meibography shortening	Meibography dropout	Corneal vascularization	Corneal pigmentation
1	7	Neutered Female	OD	10	KCS	1	1	2	2
2	12	Female	OS	7	KCS	1	0	2	2
3	10	Female	OS	5	KCS	1	1	3	2
4	10	Male	OD	7	KCS	1	1	3	0
5	9	Male	OS	6	KCS	1	1	1	2
6	10	Male	OD	6	KCS	0	1	2	0
6	10	Male	OS	6	KCS	0	1	2	0
7	11	Female	OS	5	KCS	1	1	2	2
8	13	Neutered Female	OD	5	KCS	0	1	3	1
8	13	Neutered Female	OD	5	KCS	0	1	2	0
9	12	Neutered Female	OD	6	KCS	0	0	2	0
10	11	Neutered Male	OS	6	KCS	1	0	1	2
11	12	Male	OS	3	KCS	2	0	2	0
12	10	Neutered Female	OS	3	KCS	1	0	1	2
13	15	Male	OD	21	Normal	0	1	0	1
13	15	Male	OS	20	Normal	1	0	0	1
14	15	Male	OD	22	Normal	0	0	0	0
15	13	Female	OS	22	Normal	1	0	0	0
16	9	Female	OD	20	Normal	0	0	0	0
16	9	Female	OS	18	Normal	0	0	0	0
17	13	Neutered Female	OD	20	Normal	0	0	0	1
17	13	Neutered Female	OS	20	Normal	0	0	0	1
18	9	Neutered Female	OD	20	Normal	0	0	0	0
19	9	Neutered Male	OD	20	Normal	0	0	0	0
20	12	Neutered Male	OD	25	Normal	1	0	0	1
20	12	Neutered Male	OS	25	Normal	1	0	0	1
21	11	Neutered Female	OD	20	Normal	1	0	0	0
21	11	Neutered Female	OS	20	Normal	1	0	0	0
22	11	Neutered Female	OD	19	Normal	1	0	0	0
22	11	Neutered Female	OS	23	Normal	1	0	0	0
23	11	Male	OD	20	Normal	1	0	0	0
23	11	Male	OS	25	Normal	0	0	0	0
24	11	Female	OD	18	Normal	0	0	0	0
24	11	Female	OS	20	Normal	0	1	0	0
25	12	Neutered Male	OD	20	Normal	0	0	0	0
25	12	Neutered Male	OS	18	Normal	0	0	0	0
26	10	Female	OD	20	Normal	1	0	0	0
26	10	Female	OS	20	Normal	1	1	0	0
27	8	Male	OD	20	Normal	0	1	0	0
27	8	Male	OS	20	Normal	0	0	0	0
28	11	Male	OD	22	Normal	1	0	0	0
28	11	Male	OS	23	Normal	1	0	0	0

The prevalence of MG shortening and dropout detected by meibography was determined for both groups, and the differences in these findings between the two groups were assessed with Fisher’s exact test. A *P* value of <0.05 was considered statistically significant.

## 3 RESULTS

**Figure 1:**
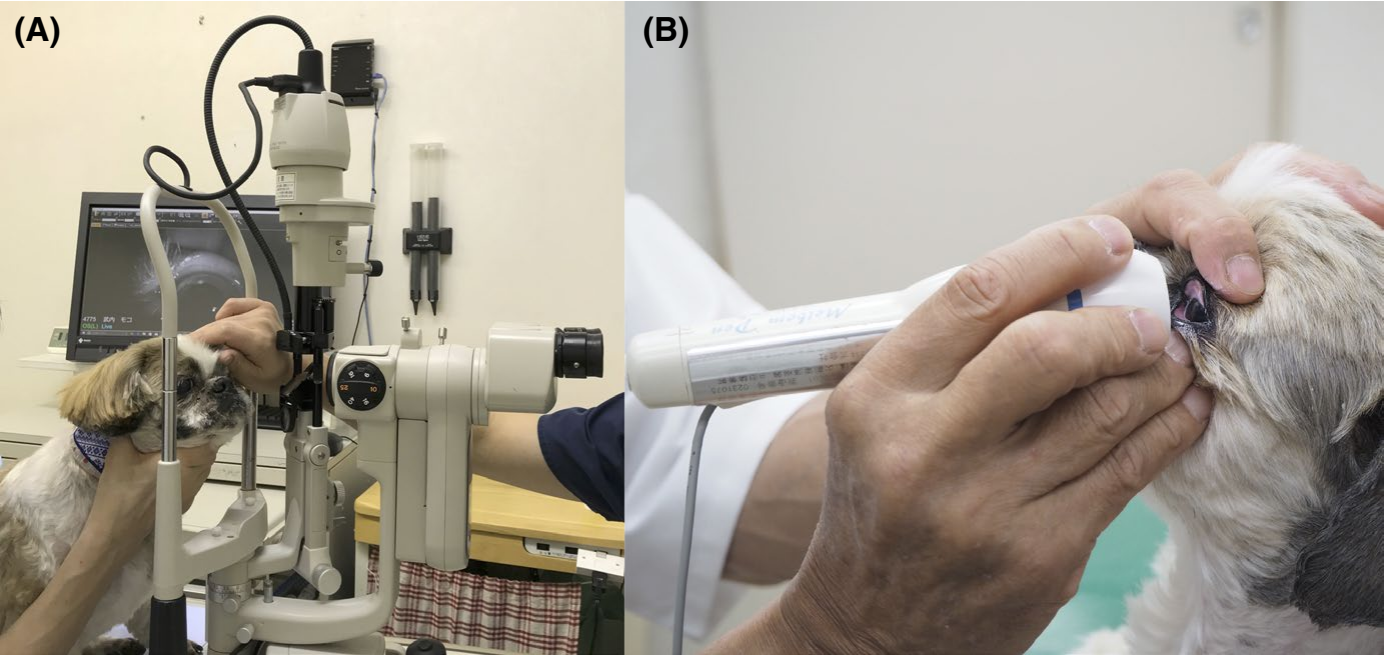
Examination of a Shih Tzu dog with a desktop meibography system (A) and portable meibography (B). The dog was placed on an examination table, and the upper eyelids were rotated outward

**Figure 2:**
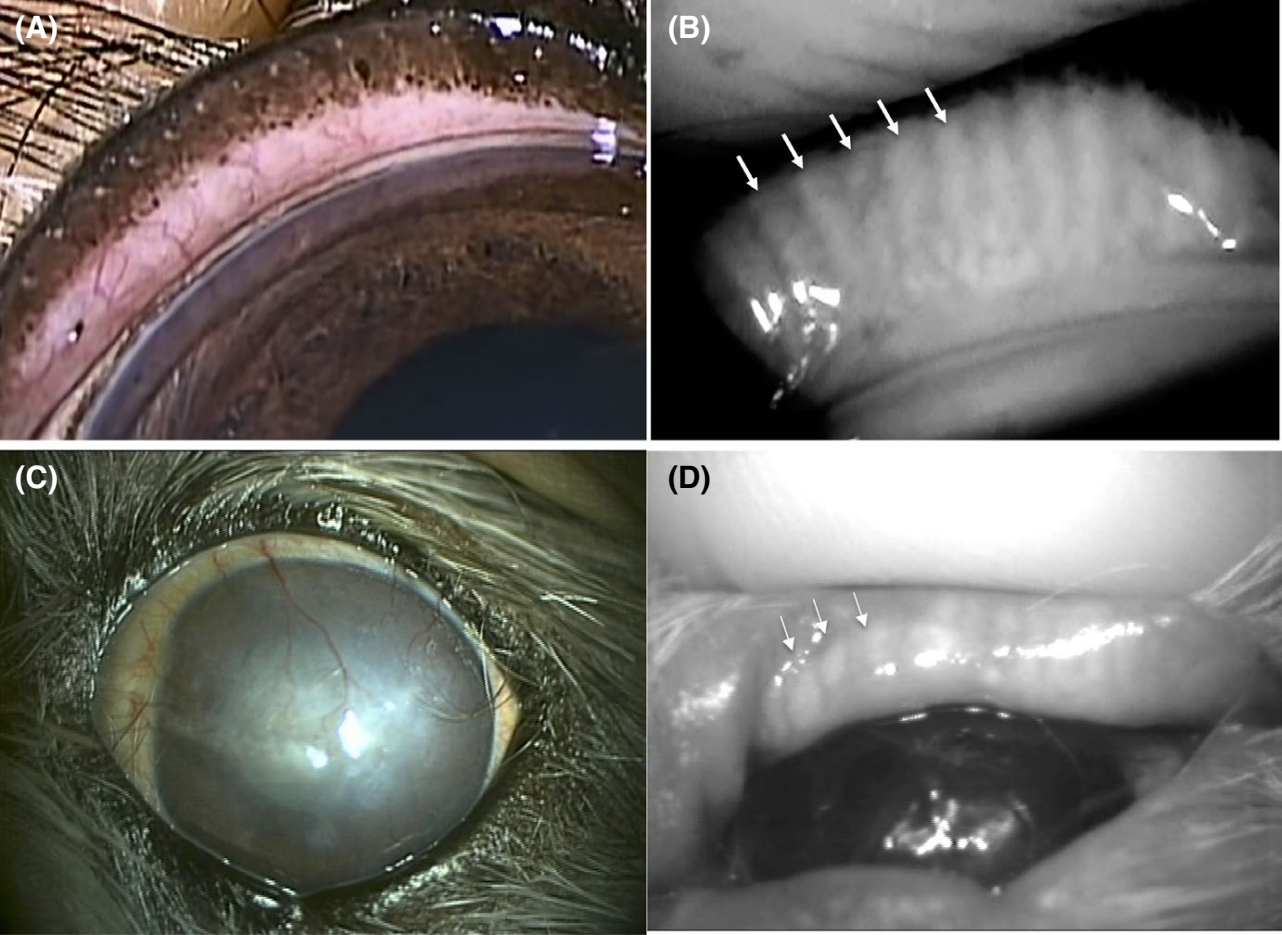
Eyelid and meibographic findings for a dog of control group. Slitlamp findings of the externally rotated eyelid. (A) MG are not apparent. Meibographic findings using desktop meibography. (B) MG can be clearly observed (arrows). Corneal and meibographic findings for a dog of KCS group Extensive corneal vascularization (C), as well as meibographic findings (D) using portable type meibography, shortening findings (arrow head) are apparent

**Figure 3:**
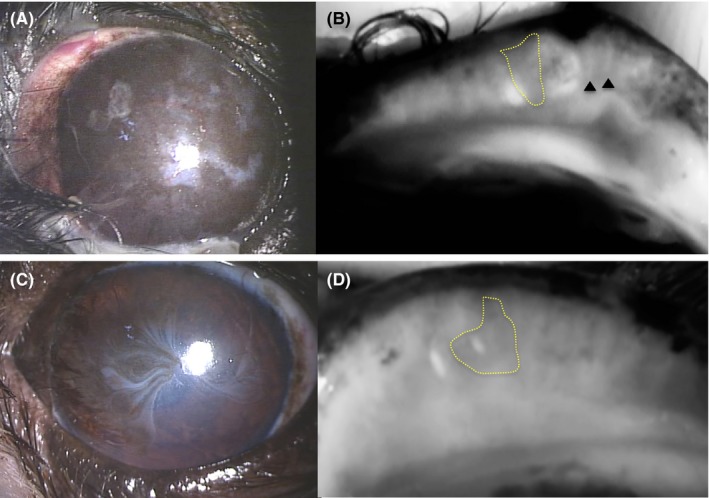
Corneal and meibographic findings for two animals of the KCS group. Extensive corneal pigmentation and vascularization, (A) as well as meibographic findings using desktop‐type meibography of gland dropout (area surrounded by dotted line) and shortening (arrowheads) (B) are apparent. Corneal vascularization (C) and the meibographic finding using desktop‐type meibography of gland dropout (area surrounded by the solid line) in the center of the eyelid (D) are apparent

**Figure 4:**
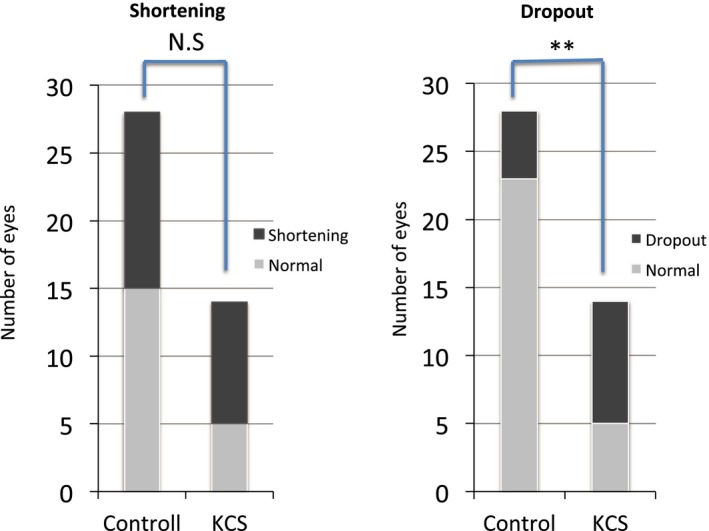
Numbers of eyes with MG shortening or dropout in the control and KCS groups. Dropout was significantly more common in the KCS group than in the control group. **P < 0.01 (Fisher’s exact test); NS, not significant

The KCS group included 14 eyes of 12 dogs with a low STT value ≤10 mm/min, mean ± SD of 6.0 ± 2.1 mm/min, and the control group included 28 eyes of 16 dogs with a normal STT value ≥15 mm/min, mean ± SD of 20.7 ± 2.4 mm/min). The findings for each eye are presented in Table 1. The KCS group included 4 intact males, 1 neutered male, 3 intact females, and 4 spayed females. The control group included 5 intact males, 3 neutered males, 4 intact females, and 4 spayed females. Examination of all eyes with both desktop and mobile noncontact infrared meibography systems was achieved by manual restraint of each animal (Figure 1). As we found previously,^8^ MGs were observed clearly by noncontact infrared meibography. Moreover, morphological abnormalities of MGs including shortening and dropout were also detected by noncontact infrared meibography (Figures 2, 3). In the KCS group, 13 of the 14 eyes showed abnormal meibography findings, with eight eyes manifesting grade 1 shortening and one eyes showing grade 2 shortening and nine eyes manifesting dropout of grade 1(Table 1). On the other hand, in the control group, morphological abnormalities were detected in 16 of the 28 eyes, with grade 1 shortening being apparent in 13 eyes and dropout in five eyes. The prevalence of dropout in the KCS group was significantly higher than that in the control group (*P* < 0.01) (Figure 4). Both types of meibography system applied yielded identical results.

## 4 DISCUSSION

MGs are sebaceous glands located in the tarsal plate of the upper and lower eyelids, and lipids secreted by MGs form the lipid layer of the tear film. Given that it is difficult to evaluate the oily layer of the tear film in veterinary ophthalmologic practice, MGs in dogs are usually examined with the use of a slitlamp to detect obstruction of gland orifices running along the limbus palpebralis as well as to determine the transparency and viscosity of the glandular lipid secreted in response to the application of pressure.^9^ Although a method based on measurement of the basal secretion volume of lipids with the use of a meibometer has been described as a functional test of MGs in dogs,^10^ problems regarding the reproducibility of such measurements have prevented the widespread application of this method.^11^ In addition, the observation of MG morphology by conventional meibography, in which the light source probe is placed in direct contact with the skin side of the eyelids,^12^, ^13^, ^14^ has not been widely adopted in veterinary ophthalmologic practice. In the present study, by manually restraining the head, we were able to rapidly examine MGs in the upper eyelids of Shih Tzu dogs with the use of both desktop and mobile types of noncontact infrared meibography system. The quality of MG images provided by portable meibography systems has previously been found to be similar to that of those acquired by desktop‐type systems,^2^ and we were able to clearly observe MG structure with both systems. Our results thus suggest that noninvasive infrared meibography is readily applicable to dogs and could be incorporated into routine ophthalmologic testing. In human medicine, noncontact meibography is used to subjectively assess MG morphology, so the area of MG loss on images is quantified. This facilitates the diagnosis of MG dysfunction (MGD) and ocular surface disease. Arita et al used a 4‐point scale from 0 to 3 points to grade the extent of MG loss in the upper and lower eyelids, resulting in what they termed the MG score.^1^ In veterinary medicine, there appear to be no studies that have described the grading of meibography images. In our study, we used noncontact meibography and the scoring system of Arita et al to grade meibography images in order to assess changes in MG morphology in dogs with or without KCS.

We compared abnormal morphological findings of MGs between dogs with KCS and control animals. We thus found that the prevalence of MG dropout was significantly higher in eyes of the KCS group than in those of the control group. We previously applied noncontact infrared meibography to examine the morphology of MGs in apparently healthy Shih Tzu dogs aged from 4 months to 15 years.^8^ We divided the dogs into 3 groups on the basis of age <3 years old (young group), 3‐10 years old (middle‐aged group), or >10 years old (elderly group)—and we found that the prevalence of MG abnormalities was significantly higher in the elderly group than in the young group, suggesting that morphological abnormalities of MGs increase with age. In the present study, given that the mean age of dogs in both the KCS and control groups was >10 years, age may have contributed to the MG morphological abnormalities observed in both groups. However, the significantly higher prevalence of gland dropout in the KCS group suggested that the pathology of KCS might also have adversely affected MG morphology.

Arita et al reported that the MG score of patients with MGD increases with age.^1^ Thus, complete or partial loss of the MGs would presumably be readily evident as an extensive change in MG morphology in patients with KCS. However, 12 eyes of patients with KCS in whom complete or partial loss of the MGs was noted were classified as Grade 1, except for 1 eye that was classified as Grade 2. Localized changes were often noted. In our study, few animals with KCS had extensive changes in MG morphology, and the reason for this is unclear. However, one possible reason is that chronic inflammation may take a prolonged period of time to cause extensive changes in MG morphology.

MGs secrete lipids via a holocrine mechanism, whereby differentiated cells in MG lobules degenerate and release their lipid contents into the gland ducts, from where they are deposited at the ocular surface through the action of blinking. In our previous study,^8^ we compared abnormal findings of meibography with histopathologic analysis. Histological staining revealed that shortening of MGs as detected by meibography was associated with loss of lobular structure and the presence of multiple cysts likely reflecting ductal hyperplasia. In addition, the histological correlates of gland dropout included disintegration of the lobular structure and ducts of MGs as well as the presence of empty vacuole‐like structures within the connective tissue. Although we did not perform a histopathologic examination in the present study, our previous findings thus suggest that the morphological abnormalities detected in eyes with KCS would result in impairment of lipid secretion by MGs. However, noncontact meibography is a means of assessing MG morphology, and cannot detect quality changes in MGs, therefore other examinations or equipment would be needed to examine MG function.

KCS is characterized by quantitative changes in the tear film and acute or chronic inflammation of the conjunctiva and cornea due to activation of the innate and adaptive immunity. Persistence of inflammation over a long period can lead to its spread to the limbus palpebralis and skin surrounding the eye.^15^ Given that the orifices of MGs lie within the limbus palpebralis, such inflammation can result in changes to the gland orifices and the tissue surrounding them and in consequent stagnation of their lipid secretory contents. In humans, the chronic obstruction of MG orifices and changes to secretory lipids are thought to lead to dilatation of MG ducts and atrophy of MG acini.^16^ In the present study, although we did not examine MG orifices and MG lipid properties in detail, the morphological abnormalities detected by meibography and the inflammatory findings of the limbus palpebralis observed by slitlamp in eyes affected by KCS are likely related.

Meibomian gland dysfunction (MGD) is diagnosed in humans on the basis of diffuse MG hypofunctionality as revealed by morphological and functional tests. Treatments for MGD include the administration of oily eyedrops or ointment as well as the application of a hot compress to the eyelids in order to improve MG secretion by melting the pathologically altered meibomian lipids.^17^, ^18^, ^19^, ^20^, ^21^, ^22^, ^23^, ^24^, ^25^, ^26^ Although we did not test MG function in the present study, meibographic examination revealed that morphological changes to MGs were more common in dogs with KCS compared with control animals. The former animals may thus have experienced the accumulation of stagnant lipids secreted by MGs. The treatment of KCS in dogs includes the administration of anti‐inflammatory agents to increase the secretion of lacrimal fluid collaterally by reactivation of functional lacrimal glands tissue. However, our results suggest that further studies are warranted to determine whether treatment to promote meibum secretion or supplementation with components of the lipid layer of the tear film might also be effective for lubrication of the ocular surface to promote smooth blinking in dogs with KCS.

The limitations of the present study include its cross‐sectional design and the fact that the duration of KCS in the subjects was not known. In addition, the study included only Shih Tzu dogs, a brachycephalic breed that is popular in Japan and in which ocular surface disease is relatively common. Morphological changes to MGs in humans are influenced by various factors including age, eye disease, and sex.^27^ The prevalence of MGD, a major cause of dry eye, is 3‐20 times higher in Asians than in Caucasian individuals.^28^, ^29^, ^30^, ^31^, ^32^, ^33^, ^34^ Furthermore, acinar cells in MGs have been found to be affected by steroid hormones such as androgens.^35^, ^36^ The neutering of dogs by spaying or castration might therefore affect the morphology of MGs. Further studies are thus warranted to examine the possible effects of sex and breed on MG abnormalities in dogs.
